# Impact of Partial Slip on Double Diffusion Convection of Sisko Nanofluids in Asymmetric Channel with Peristaltic Propulsion and Inclined Magnetic Field

**DOI:** 10.3390/nano12162736

**Published:** 2022-08-09

**Authors:** Safia Akram, Maria Athar, Khalid Saeed, Alia Razia, Metib Alghamdi, Taseer Muhammad

**Affiliations:** 1 MCS, National University of Sciences and Technology, Islamabad 44000, Pakistan; 2 Department of Mathematics, National University of Modern Languages, Islamabad 44000, Pakistan; 3 Department of Mathematics, COMSATS University, Islamabad 45550, Pakistan; 4 Department of Mathematics, College of Sciences, King Khalid University, Abha 61413, Saudi Arabia

**Keywords:** partial slip, Sisko fluid, double diffusion convection, inclined magnetic field, asymmetric channel, peristaltic flow

## Abstract

The current article discusses the outcomes of the double diffusion convection of peristaltic transport in Sisko nanofluids along an asymmetric channel having an inclined magnetic field. Consideration is given to the Sisko fluid model, which can forecast both Newtonian and non-Newtonian fluid properties. Lubricating greases are the best examples of Sisko fluids. Experimental research shows that most realistic fluids, including human blood, paint, dirt, and other substances, correspond to Sisko’s proposed definition of viscosity. Mathematical modelling is considered to explain the flow behavior. The simpler non-linear PEDs are deduced by using an elongated wavelength and a minimal Reynolds number. The expression is also numerically calculated. The impacts of the physical variables on the quantities of flow are plotted graphically as well as numerically. The results reveal that there is a remarkable increase in the concentration, temperature, and nanoparticle fraction with the rise in the Dufour and thermophoresis variables.

## 1. Introduction

The study of non-Newtonian models is the pinnacle of contemporary research owing to the fact that it has tremendous practical utility in the manufacturing field. The common domains are electrical engineering or electricity generation, the energy industry, heat–power engineering, and the chemical and pharmaceutical industry. Moreover, many studies have focused on the application of non-Newtonian fluids in the biomedical industry, environmental management, and catalysis science and technology. The classification of non-Newtonian fluids is quite broad and one can find various categories of models because exclusive intrinsic relation to any category is insufficient to comprehensively elaborate all the properties of these fluids. The Sisko model is notable among all models because the constitutive equation of the model is exhaustive enough to reveal the fluid’s behavior. It can elaborate the shear-thinning trend in the fluid as the shear-thinning rate is low and velocity reaches the constant value when the shear rate is high. The same phenomenon is used to study the blood circulation mechanism. Sisko, in 1958 [1], developed the model with the help of the power law model of fluid that is the combination of non-Newtonian and Newtonian fluids. Naturally occurring fluids fall in this category that has many practical applications such as the transport of greasy fluids.

Peristaltic techniques and their reactions have fundamental implications in human physiology, or one can say that they are the essential mechanisms on which all human systems function. The term peristalsis is coined from the Greek word peristaltikos meaning condensing and contracting. All the muscular movements in the human body are peristaltic motions. These are involuntary muscular contractions and relaxations for the transportation of fluids between various organs, for example, for urination, and food swallowing and digestion in the gastro-intestinal tract, and other fluid transport such as chyme motion, lymph secretion and transportation, blood circulation, serum and eggs movement in the sexual organs, etc. Hence, peristalsis is a phenomenal process in physiological functioning. On the other hand, its significance is apparent in physical and biomedical sciences where the principle of peristalsis has been utilized to design equipment such as pumping machines and rollers. It has led to the envisioning of a vast area of scientific discoveries to achieve excellence in equipment and tool production. Therefore, contemporary researches have employed the mathematical models of peristalsis techniques in non-Newtonian and Newtonian fluid [2,3,4,5,6,7,8,9,10,11,12].

Another noteworthy area of peristaltic flow under magnetic flux is engineering and technical sciences. Magnetohydrodynamics (MHD) is a specialized domain governed by electromagnetic power, which includes magneto-liquids such as water, plasma, and electrolytes. The main branch of MHD is resonance imaging where it has been used to assist medical procedures such as to control blood loss in surgery, the spreading of cancer cells and the proliferation of magnetic tracers, and the study of blood circulation and pumping in the intestinal tract and glands’ ducts. The different methods of the impact of MHD on peristalsis are given in References [13,14,15,16,17,18,19,20].

The flow, called the no slip condition, is a phenomenon where the velocity of the fluid comes in proximity with the velocity of a solid that is the same as the boundary velocity. In contrast, there are some situations where the no slip condition is not applicable such as in permeable walls, suspensions, rough or coated surfaces, emulsions, polymer solutions, foam, and gases; hence, partial slip boundary conditions are pertinent to be used. Navier [21] was the first to introduce and use this concept. Later on, the idea was further explored and has been extended with various geometries by many researchers [22,23,24,25,26,27,28].

In recent investigations, the phenomenon of peristalsis was investigated for the domain of nanofluids. Liquids with tiny, suspended particles, less than the 1000th width of human hair, are called nanofluids. These liquids have the tendency of high heat transfer when compared to any other liquid. Therefore, these are important for heat transfer procedures such as in a nuclear furnace, the electron industry, biomedicine, food processing, and transportation, as well as the help in cooling processes used in the welding industry and automobile corporation. Choi [29] was the pioneer to apply the concept of nanofluids. Then, following in his footsteps, many investigators expanded the area with the help of differing geometries [30,31,32,33,34,35,36,37,38,39,40,41,42,43,44,45,46].

The flow, geared by the buoyancy of complementing concentration and temperature gradients, is called double diffusive natural convection. The phenomenon can be observed in nature as the flow of gases in the atmosphere, and water flow in oceans, coastal regions, lakes, etc. It also has wide industrial utilization in crystallization, the physical processing of various materials, and energy repositories. Significant investigations were performed by Ostrach [47] and Viskanta et al. [48], while other relevant studies are listed in References [49,50,51,52,53,54].

Based on the compelling industrial utilization as mentioned in the above referred literature, the present paper attempts to theoretically examine the slip boundary impact on double diffusive peristaltic nanofluid flow using Sisko fluid as a base along an asymmetric channel under an inclined magnetic flux. Nanofluids with a magnetic flux have many industrial applications, mainly in optics to manufacture optical switches, wavelength filters, modulators, and fiber filters. Moreover, these are important in the biomedical manufacturing industries, therapeutical treatment of cancer, and floating dividers. Since magnetic nanoparticles are stickier and more cohesive towards cancer tissues than any normal cell, they are used to carry drug particles to the tumor-infected region through the blood stream. They have more power to absorb heat than any other microparticle; hence, they are used to change the magnetic current field within the human body that is required in cancer therapy. Contrast magnetic resonance imaging, hyperthermia, therapeutic procedures, and drug transfer are some of the other significant fields of application. The layout of this paper is as follows:

The motion equation along with the Sisko fluid equation is discussed in Section 2. Formulation of the problem is in Section 3, which also reflects on the governing problem in a linear fashion. Section 4 covers the numerical and graphical results and its explanation, while the last section is a comprehensive conclusion based on the main findings.

## 2. Mathematical Formulation

Let us consider peristaltic flux in non-Newtonian fluid in an asymmetric conduit with continual magnetic field. The assumptions are that the wave line on channel walls is moving with velocity c, and the angle of magnetic field is inclined at ω. A uniform magnetic field $B=(B_{0}sin\omega ,$ $B_{0}cos\omega ,$ 0) is imposed. When considering minute magnetic Reynolds number, the induced magnetic field is neglected. Moreover, the $y=H_{1}$ (top wall) and $y=H_{2}$ (lower wall) are held at temperature, concentration, and solute of nanoparticles with $T=T_{0},$ $C=C_{0},$ $\Theta =\Theta_{0}$, and $T=T_{1},$ $C=C_{1},$ $\Theta =\Theta_{1}$, respectively.

**Figure 1:**
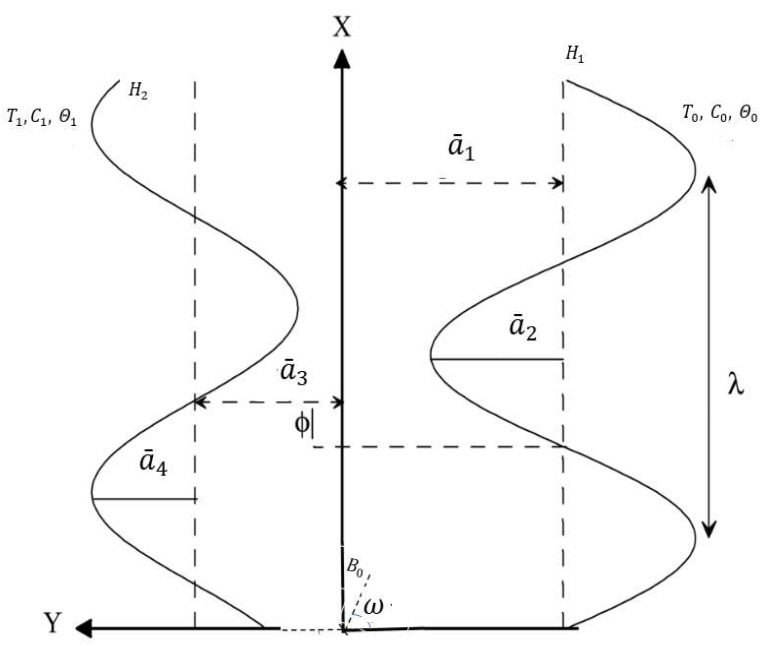
Geometry of the problem.

The structure of a wall is sketched in Figure 1, and mathematically it is defined as [19]

(1) $$Y=H_{1}={\bar{a}}_{1}+{\bar{a}}_{2}cos\left(\left(X-ct\right)\frac{2\pi }{\lambda }\right),\;\;Y=H_{2}=-{\bar{a}}_{3}-{\bar{a}}_{4}cos\left(\left(X-ct\right)\frac{2\pi }{\lambda }+\phi \right),\;$$

Here X stands for wave propagation direction, c represents velocity propagation, $({\bar{a}}_{2},$ ${\bar{a}}_{4})$ act as wave amplitudes, ${\bar{a}}_{1}+{\bar{a}}_{3}$ is channel width, λ denotes wavelength, t represents time, The phase difference ϕ range is 0≤ϕ≤π, ϕ=0 implies a symmetric channel, without waves phase, and ϕ=π indicates a channel with in waves phase. In addition, ${\bar{a}}_{1},$ ${\bar{a}}_{2},$ ${\bar{a}}_{3},$ ${\bar{a}}_{4}$, and ϕ satisfy the constraint ${\bar{a}}_{2}^{2}+{\bar{a}}_{4}^{2}+2{\bar{a}}_{2}{\bar{a}}_{4}cos\phi \leq {\left({\bar{a}}_{1}+{\bar{a}}_{3}\right)}^{2}.$

The Sisko fluid stress tensor is defined by [1]

(2) $$S=\left(\tilde{\eta }+\bar{\beta }{\left(\sqrt{\Pi }\right)}^{i-1}\right)A_{1},\;A_{1}=L+L^{T},\;L=gradV,\;\Pi =\frac{1}{2}trac\left(A_{1}^{2}\right),\;$$

The velocities U and V along X and Y are taken for the current flow, so the continuity equation, momentum, temperature, nanoparticles fraction, and the solute concentration of an incompressible fluid at a fixed frame, is given as

(3) $$\frac{\partial U}{\partial X}+\frac{\partial V}{\partial Y}=0,\;$$

(4) $$\rho_{f}\left(\frac{\partial }{\partial t}+U\frac{\partial }{\partial X}+V\frac{\partial }{\partial Y}\right)U=-\frac{\partial P}{\partial X}+\frac{\partial S_{XX}}{\partial X}+\frac{\partial S_{XY}}{\partial Y}-\sigma B_{0}^{2}cos\omega \left(Ucos\omega -Vsin\omega \right)\;+g\rho_{f}\{\left(1-\Theta_{0}\right)\rho_{f0}\left\{\beta_{T}\left(T-T_{0}\right)+\beta_{C}\left(C-C_{0}\right)\right\}-\left(\rho_{p}-\rho_{f0}\right)\left(\Theta -\Theta_{0}\right)\},\;$$

(5) $$\rho_{f}\left(\frac{\partial }{\partial t}+U\frac{\partial }{\partial X}+V\frac{\partial }{\partial Y}\right)V=-\frac{\partial P}{\partial Y}+\frac{\partial S_{YX}}{\partial X}+\frac{\partial S_{YY}}{\partial Y}+\sigma B_{0}^{2}sin\omega \left(Ucos\omega -Vsin\omega \right),\;$$

(6) $$\begin{array}{l} {\left(\rho c\right)}_{f}\left(\frac{\partial }{\partial t}+U\frac{\partial }{\partial X}+V\frac{\partial }{\partial Y}\right)T=k\left(\frac{\partial^{2}T}{\partial X^{2}}+\frac{\partial^{2}T}{\partial Y^{2}}\right)+{\left(\rho c\right)}_{p}\{D_{B}\left(\frac{\partial T}{\partial X}\frac{\partial \Theta }{\partial X}+\frac{\partial T}{\partial Y}\frac{\partial \Theta }{\partial Y}\right)\\ \left(\frac{D_{T}}{T_{0}}\right)\left[{\left(\frac{\partial T}{\partial X}\right)}^{2}+{\left(\frac{\partial T}{\partial Y}\right)}^{2}\right]\}+D_{TC}\left(\frac{\partial^{2}C}{\partial X^{2}}+\frac{\partial^{2}C}{\partial Y^{2}}\right),\; \end{array}$$

(7) $$\left(\frac{\partial }{\partial t}+U\frac{\partial }{\partial X}+V\frac{\partial }{\partial Y}\right)C=D_{s}\left(\frac{\partial^{2}C}{\partial X^{2}}+\frac{\partial^{2}C}{\partial Y^{2}}\right)+D_{TC}\left(\frac{\partial^{2}T}{\partial X^{2}}+\frac{\partial^{2}T}{\partial Y^{2}}\right),\;$$

(8) $$\left(\frac{\partial }{\partial t}+U\frac{\partial }{\partial X}+V\frac{\partial }{\partial Y}\right)\Theta =D_{B}\left(\frac{\partial^{2}\Theta }{\partial X^{2}}+\frac{\partial^{2}\Theta }{\partial Y^{2}}\right)+\left(\frac{D_{T}}{T_{0}}\right)\left(\frac{\partial^{2}T}{\partial X^{2}}+\frac{\partial^{2}T}{\partial Y^{2}}\right),\;$$

where component form of stresses is defined as

(9) $$\begin{array}{l} S_{XX}=2\frac{\partial U}{\partial X}\left(\tilde{\eta }+\bar{\beta }{\left\{2{\left(\frac{\partial U}{\partial X}\right)}^{2}+{\left(\frac{\partial U}{\partial Y}+\frac{\partial V}{\partial X}\right)}^{2}+2{\left(\frac{\partial V}{\partial Y}\right)}^{2}\right\}}^{\frac{i-1}{2}}\right),\\ S_{XY}=\left(\frac{\partial U}{\partial Y}+\frac{\partial V}{\partial X}\right)\left(\tilde{\eta }+\bar{\beta }{\left\{2{\left(\frac{\partial U}{\partial X}\right)}^{2}+{\left(\frac{\partial U}{\partial Y}+\frac{\partial V}{\partial X}\right)}^{2}+2{\left(\frac{\partial V}{\partial Y}\right)}^{2}\right\}}^{\frac{i-1}{2}}\right),\\ S_{YY}=2\frac{\partial V}{\partial Y}\left(\tilde{\eta }+\bar{\beta }{\left\{2{\left(\frac{\partial U}{\partial X}\right)}^{2}+{\left(\frac{\partial U}{\partial Y}+\frac{\partial V}{\partial X}\right)}^{2}+2{\left(\frac{\partial V}{\partial Y}\right)}^{2}\right\}}^{\frac{i-1}{2}}\right),\; \end{array}$$

where g, k, T, $\left(\frac{d}{dt}\right)$, $\rho_{f},$ $\rho_{p},$ $\rho_{f_{0}},$ p, ${\left(\rho c\right)}_{p},$ ${\left(\rho c\right)}_{f}$, C, $\beta_{C},$ Θ, $\beta_{T}$, $D_{s},$ $D_{TC},$ $D_{B},$ $D_{CT},$ $D_{T}$ represent acceleration, thermal conductivity, temperature, material derivative, fluid density, nanoparticle mass density, fluid density at $T_{0}$, pressure, nanoparticle heat capacity, fluid heat capacity, solutal concentration, volumetric coefficient of solutal expansion, nanoparticle fraction, volumetric coefficient of thermal expansion, solutal diffusively, Dufour diffusively, Brownian diffusion, Soret diffusively, thermophoretic diffusion, respectively.

Now, using the well-known Galilean transformation leads to

(10) y=Y, x=X−ct, v=V, p(x,y)=P(X,Y,t), u=U−c, 

Dimensionless quantities are defined as

(11) $$\begin{array}{l} \bar{x}=\frac{x}{\lambda },\;d=\frac{{\bar{a}}_{3}}{{\bar{a}}_{1}},\;\bar{y}=\frac{y}{{\bar{a}}_{1}},\;\bar{v}=\frac{v}{c},\;\delta =\frac{{\bar{a}}_{1}}{\lambda },\;\bar{u}=\frac{u}{c},\;\bar{t}=\frac{ct}{\lambda },\;h_{2}=\frac{H_{2}}{{\bar{a}}_{3}},\;h_{1}=\frac{H_{1}}{{\bar{a}}_{1}},b=\frac{{\bar{a}}_{4}}{{\bar{a}}_{1}},\;\bar{p}=\frac{{\bar{a}}_{1}^{2}p}{\tilde{\eta }c\lambda },\;\\ a=\frac{{\bar{a}}_{2}}{{\bar{a}}_{1}},\;Pr=\frac{{\left(\rho c\right)}_{f}\;\upsilon }{k},\;Re=\frac{\rho_{f}c{\bar{a}}_{1}}{\tilde{\eta }},\;\upsilon =\frac{\mu }{\rho_{f}},\;Le=\frac{\upsilon }{D_{s}},M=\sqrt{\frac{\sigma }{\tilde{\eta }}}B_{0}{\bar{a}}_{1},\;N_{CT}=\frac{D_{CT}\left(T_{1}-T_{0}\right)}{\left(C_{1}-C_{0}\right)D_{s}},\;\\ N_{TC}=\frac{D_{CT}\left(C_{1}-C_{0}\right)}{k\left(T_{1}-T_{0}\right)},\;N_{t}=\frac{{\left(\rho c\right)}_{p}D_{T}\left(T_{1}-T_{0}\right)}{T_{0}k},G_{rt}=\frac{g{\bar{a}}_{1}^{2}\left(T_{1}-T_{0}\right)\left(1-\Theta_{0}\right)\rho_{f}\beta_{T}}{\tilde{\eta }c},\\ G_{rF}=\frac{g\left(\rho_{p}-\rho_{f}\right)\left(\Theta_{1}-\Theta_{0}\right)}{\tilde{\eta }c}{\bar{a}}_{1}^{2},\;\bar{S}=\frac{{\bar{a}}_{1}}{\tilde{\eta }c}S,\;Ln=\frac{\upsilon }{D_{B}},\;\theta =\frac{T-T_{0}}{T_{1}-T_{0}},\;\gamma =\frac{C-C_{0}}{C_{1}-C_{0}},\;\\ \Omega =\frac{\Theta -\Theta_{0}}{\Theta_{1}-\Theta_{0}},\;v=-\delta \frac{\partial \Psi }{\partial x},\;u=\frac{\partial \Psi }{\partial y},\beta =\frac{\bar{\beta }}{\tilde{\eta }{\left({\bar{a}}_{1}/c\right)}^{i-1}},\;N_{b}=\frac{{\left(\rho c\right)}_{p}D_{B}\left(\Theta_{1}-\Theta_{0}\right)}{k},\;\\ G_{rc}=\frac{\left(1-\Theta_{0}\right)\rho_{f}g\beta_{c}\left(C_{1}-C_{0}\right){\bar{a}}_{1}^{2}}{\tilde{\eta }c},\; \end{array}$$

Here Ω, Le, Pr, γ, $G_{rt},$ δ, $N_{CT},$ $G_{rF},$ $N_{t},$ θ, $N_{b},$ Ln, $N_{TC}$, $G_{rc}$ denote nanoparticle fraction, Lewis number, Prandtl number, solutal (species) concentration, thermal Grashof number, wave number, Soret parameter, nanoparticle Grashof number, Reynolds number, thermophoresis parameters, temperature, Brownian motion, nanofluid Lewis number, parameter of Dufour, and solutal Grashof number, respectively.

Using Equations (10) and (11), Equation (3) is satisfied automatically and Equations (4)–(9) after bars dropping in wave frame become

(12) $$\begin{array}{l} Re\delta \left(\Psi_{xy}\Psi_{y}-\Psi_{yy}\Psi_{x}\right)=-\frac{\partial p}{\partial x}+\delta \frac{\partial S_{xx}}{\partial x}+\frac{\partial S_{xy}}{\partial y}-M^{2}cos\omega \left(cos\omega \left(\Psi_{y}+1\right)+\delta sin\omega \Psi_{x}\right)\\ +G_{rt}\theta +G_{rc}\gamma -G_{rF}\Omega ,\; \end{array}$$

(13) $$Re\delta^{3}\left(\Psi_{xy}\Psi_{x}-\Psi_{xx}\Psi_{y}\right)=-\frac{\partial p}{\partial y}+\delta^{2}\frac{\partial S_{xy}}{\partial x}+\delta \frac{\partial S_{yy}}{\partial y}\;+\delta M^{2}sin\omega \left(cos\omega \left(\Psi_{y}+1\right)+\delta sin\omega \Psi_{x}\right),\;$$

(14) $$Re\delta Pr\left(\theta_{x}\Psi_{y}-\theta_{y}\Psi_{x}\right)=\left(\theta_{yy}+\delta^{2}\theta_{xx}\right)+N_{TC}\left(\delta^{2}\gamma_{xx}+\gamma_{yy}\right)+N_{b}\left(\delta^{2}\Omega_{x}\theta_{x}+\Omega_{y}\theta_{y}\right)\;+N_{t}\left(\delta^{2}{\left(\theta_{x}\right)}^{2}+{\left(\theta_{y}\right)}^{2}\right),\;$$

(15) $$ReLe\delta \left(\gamma_{x}\Psi_{y}-\gamma_{y}\Psi_{x}\right)=\left(\delta^{2}\gamma_{xx}+\gamma_{yy}\right)+N_{CT}\left(\delta^{2}\theta_{xx}+\theta_{yy}\right),\;$$

(16) $$ReLn\delta \left(\Psi_{y}\Omega_{x}-\Psi_{x}\Omega_{y}\right)=\left(\delta^{2}\Omega_{xx}+\Omega_{yy}\right)+\frac{N_{t}}{N_{b}}\left(\delta^{2}\theta_{xx}+\theta_{yy}\right),\;$$

where

(17) $$\begin{array}{l} S_{xx}=2\delta \left(1+\beta {\left\{2\delta^{2}{\left(\frac{\partial^{2}\Psi }{\partial x\partial y}\right)}^{2}+{\left(\frac{\partial^{2}\Psi }{\partial y^{2}}-\delta^{2}\frac{\partial^{2}\Psi }{\partial x^{2}}\right)}^{2}+2\delta^{2}{\left(\frac{\partial^{2}\Psi }{\partial x\partial y}\right)}^{2}\right\}}^{\frac{i-1}{2}}\right)\frac{\partial^{2}\Psi }{\partial x\partial y},\\ S_{xy}=\left(1+\beta {\left\{2\delta^{2}{\left(\frac{\partial^{2}\Psi }{\partial x\partial y}\right)}^{2}+{\left(\frac{\partial^{2}\Psi }{\partial y^{2}}-\delta^{2}\frac{\partial^{2}\Psi }{\partial x^{2}}\right)}^{2}+2\delta^{2}{\left(\frac{\partial^{2}\Psi }{\partial x\partial y}\right)}^{2}\right\}}^{\frac{i-1}{2}}\right)\left(\frac{\partial^{2}\Psi }{\partial y^{2}}-\delta^{2}\frac{\partial^{2}\Psi }{\partial x^{2}}\right),\\ S_{yy}=-2\delta \left(1+\beta {\left\{2\delta^{2}{\left(\frac{\partial^{2}\Psi }{\partial x\partial y}\right)}^{2}+{\left(\frac{\partial^{2}\Psi }{\partial y^{2}}-\delta^{2}\frac{\partial^{2}\Psi }{\partial x^{2}}\right)}^{2}+2\delta^{2}{\left(\frac{\partial^{2}\Psi }{\partial x\partial y}\right)}^{2}\right\}}^{\frac{i-1}{2}}\right)\frac{\partial^{2}\Psi }{\partial x\partial y},\; \end{array}$$

Now, using approximation of low Reynolds number and long wavelength, the Equations (12)–(17) are reduced to the form as

(18) $$-\frac{\partial p}{\partial x}+\frac{\partial S_{xy}}{\partial y}-M^{2}{cos}^{2}\omega \left(\Psi_{y}+1\right)+G_{rt}\theta +G_{rc}\gamma -G_{rF}\Omega =0,\;$$

(19) $$-\frac{\partial p}{\partial y}=0,\;$$

(20) $$\frac{\partial^{2}\theta }{\partial y^{2}}+N_{TC}\frac{\partial^{2}\gamma }{\partial y^{2}}+N_{b}\left(\frac{\partial \Omega }{\partial y}\frac{\partial \theta }{\partial y}\right)+N_{t}{\left(\frac{\partial \theta }{\partial y}\right)}^{2}=0,\;$$

(21) $$\frac{\partial^{2}\gamma }{\partial y^{2}}+N_{CT}\frac{\partial^{2}\theta }{\partial y^{2}}=0,\;$$

(22) $$\frac{\partial^{2}\Omega }{\partial y^{2}}+\frac{N_{t}}{N_{b}}\frac{\partial^{2}\theta }{\partial y^{2}}=0,\;$$

where

(23) $$S_{xy}=\left(1+\beta {\left\{{\left(\frac{\partial^{2}\Psi }{\partial y^{2}}\right)}^{2}\right\}}^{\frac{i-1}{2}}\right)\frac{\partial^{2}\Psi }{\partial y^{2}},\;$$

By eliminating the pressure from Equations (18) and (19), we obtained the following equations

(24) $$\frac{\partial^{2}}{\partial y^{2}}\left[1+\beta {\left\{{\left(\frac{\partial^{2}\Psi }{\partial y^{2}}\right)}^{2}\right\}}^{\frac{i-1}{2}}\right]\frac{\partial^{2}\Psi }{\partial y^{2}}-M^{2}{cos}^{2}\omega \frac{\partial^{2}\Psi }{\partial y^{2}}+G_{rt}\frac{\partial \theta }{\partial y}+G_{rc}\frac{\partial \gamma }{\partial y}-G_{rF}\frac{\partial \Omega }{\partial y}=0,\;$$

(25) $$\frac{\partial^{4}\Psi }{\partial y^{4}}+\beta \frac{\partial^{2}}{\partial y^{2}}{\left\{\left(\frac{\partial^{2}\Psi }{\partial y^{2}}\right)\right\}}^{i}-M^{2}{cos}^{2}\omega \frac{\partial^{2}\Psi }{\partial y^{2}}+G_{rt}\frac{\partial \theta }{\partial y}+G_{rc}\frac{\partial \gamma }{\partial y}-G_{rF}\frac{\partial \Omega }{\partial y}=0,\;$$

The existing problem boundary conditions are expressed in wave frame as follows

(26) $$\begin{array}{l} \Psi =\frac{\hat{F}}{2},\;\frac{\partial \Psi }{\partial y}+\Gamma_{1}\;S_{xy}=-1\;\mathrm{at}\;y=h_{1}\left(x\right),\\ \Psi =-\frac{\hat{F}}{2},\;\frac{\partial \Psi }{\partial y}-\Gamma_{1}\;S_{xy}=-1\;\mathrm{at}\;y=h_{2}\left(x\right),\; \end{array}$$

(27) $$\theta +\Gamma_{2}\;\frac{\partial \theta }{\partial y}=0,\;\mathrm{at}\;y=h_{1},\;\mathrm{and}\;\theta -\Gamma_{2}\;\frac{\partial \theta }{\partial y}=1,\;\mathrm{at}\;y=h_{2},\;$$

(28) $$\gamma +\Gamma_{3}\;\frac{\partial \gamma }{\partial y}=0,\;\mathrm{at}\;y=h_{1},\;\mathrm{and}\;\gamma -\Gamma_{3}\;\frac{\partial \gamma }{\partial y}=1,\;\mathrm{at}\;y=h_{2},\;$$

(29) $$\Omega +\Gamma_{4}\;\frac{\partial \Omega }{\partial y}=0,\;\mathrm{at}\;y=h_{1},\;\mathrm{and}\;\Omega -\Gamma_{4}\;\frac{\partial \Omega }{\partial y}=1,\;\mathrm{at}\;y=h_{2},\;$$

The case of no slip boundary conditions exists when we consider $\Gamma_{1},$ $\Gamma_{2},$ $\Gamma_{3},$ $\Gamma_{4}=0$ in Equations (27)–(30).

The flow rate Q in dimensionless form is represented as [19]

(30) $$Q=1+d+\hat{F}.\;$$

where

(31) $$\hat{F}=\int_{h_{2}\left(x\right)}^{h_{1}\left(x\right)}\frac{\partial \Psi }{\partial y}dy=\Psi \left(h_{1}\left(x\right)-h_{2}\left(x\right)\right),\;$$

here

(32) $$h_{1}\left(x\right)=1+acos2\pi x,\;h_{2}\left(x\right)=-d-bcos(2\pi x+\phi ).\;$$

### Special Case

In the non-existence of slip conditions $\left(\Gamma_{1}=\Gamma_{2}=\Gamma_{3}=\Gamma_{4}=0\right)$, $M=0,\;\;G_{rt}=0,\;G_{rF}=0,\;G_{rc}=0,$ the results of Mishra and Rao [3] can be recovered as a special case of our problem.

## 3. Numerical Solution and Graphical Outcomes

Due to the non-linear system and coupled behavior, exact solutions to the systems of PDEs (18), (20)–(22), and (25) are difficult to find. As a result, we can use Mathematica’s NDSolve command to find the numerical solutions to Equations (18), (20)–(22), and (25) as well as the boundary conditions (26)–(29). NDSolve is a Wolfram language function that solves numerical differential equations in a broad sense. In addition to various ordinary differential equations, it can handle a variety of partial differential equations. This command uses interpolating function objects to iteratively find the solutions. As a result of the numerical solution, the impact of the evolving parameters of fluid quantities is examined via graphical illustration.

### 3.1. Effects of Hartmann Number (M)

**Figure 2:**
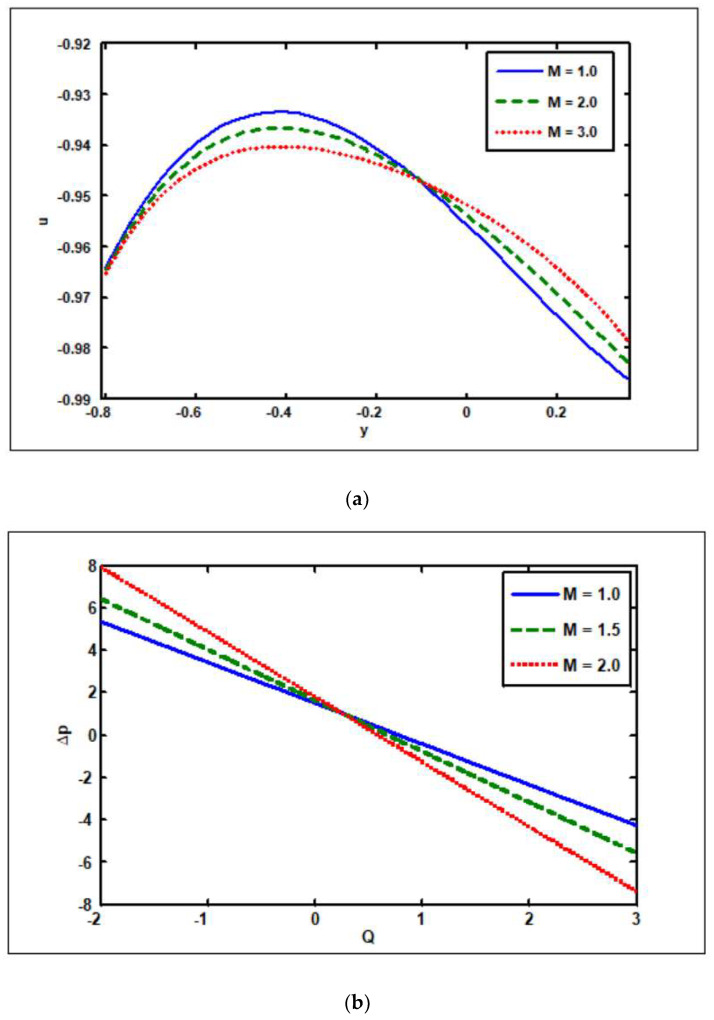

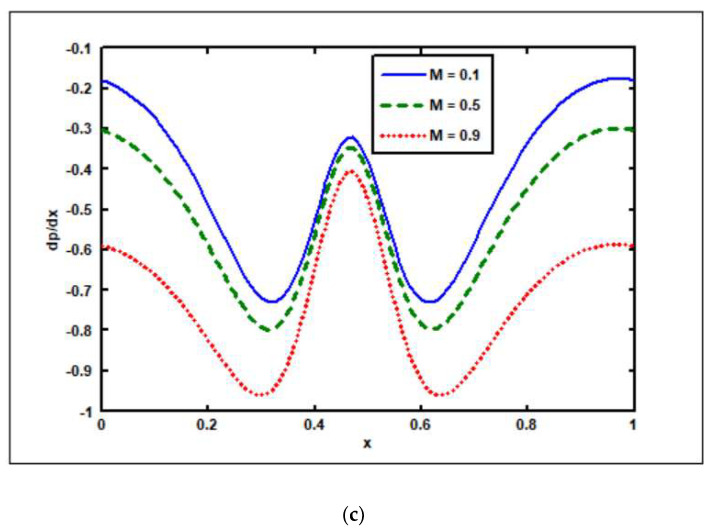
Hartmann number’s, M, impact on (**a**) velocity, (**b**) pressure rise, and (**c**) pressure gradient. Other parameters’ values are as follows: $G_{rc}=1.4,\;N_{CT}=0.9,\;N_{TC}=1.2,\;\beta =0.1,\;\Gamma_{1}=0.2,\;\Gamma_{2}=0.4,\;\Gamma_{3}=0.3,\;i=2,\;\Gamma_{4}=0.5,\;G_{rF}=0.8,\;G_{rt}=0.5,\;N_{b}=0.4,\;N_{t}=0.3,\;Q=1,\;\omega =\pi /5,\;\varphi =\pi /4,\;a=0.8,\;b=0.3,\;d=1.1$.

The plots in Figure 2a–c are drawn to see how the Hartmann number (M) affects the velocity, pressure gradient, and pressure rise. In the interval when y∈[−0.8,−0.1], the velocity magnitude increases as M becomes larger, while in the interval when y∈[−0.1,0.1], the reverse trend is observed (see Figure 2a). This occurs because as the magnetic number grows, the Lorentz force, which acts as a retarding force, increases, resulting in the fluid motion decelerating. The characteristic of pressure rises on M is depicted in Figure 2b. It is obvious that escalating M enhances the pressure rise of the retrograde zone (Δp>0,Q<0), peristaltic zone (Δp>0,Q>0), and free (Δp=0) pumping segment, while increasing M lessens the pressure rise in the augmented (Q>0,Δp<0) region. As the Hartmann number rises, the pressure gradient magnitude goes up as well (see Figure 2c).

### 3.2. Effects of Slip Parameters ($\Gamma_{1},$ $\Gamma_{2},$ $\Gamma_{3},$ $\Gamma_{4}$)

**Figure 3:**
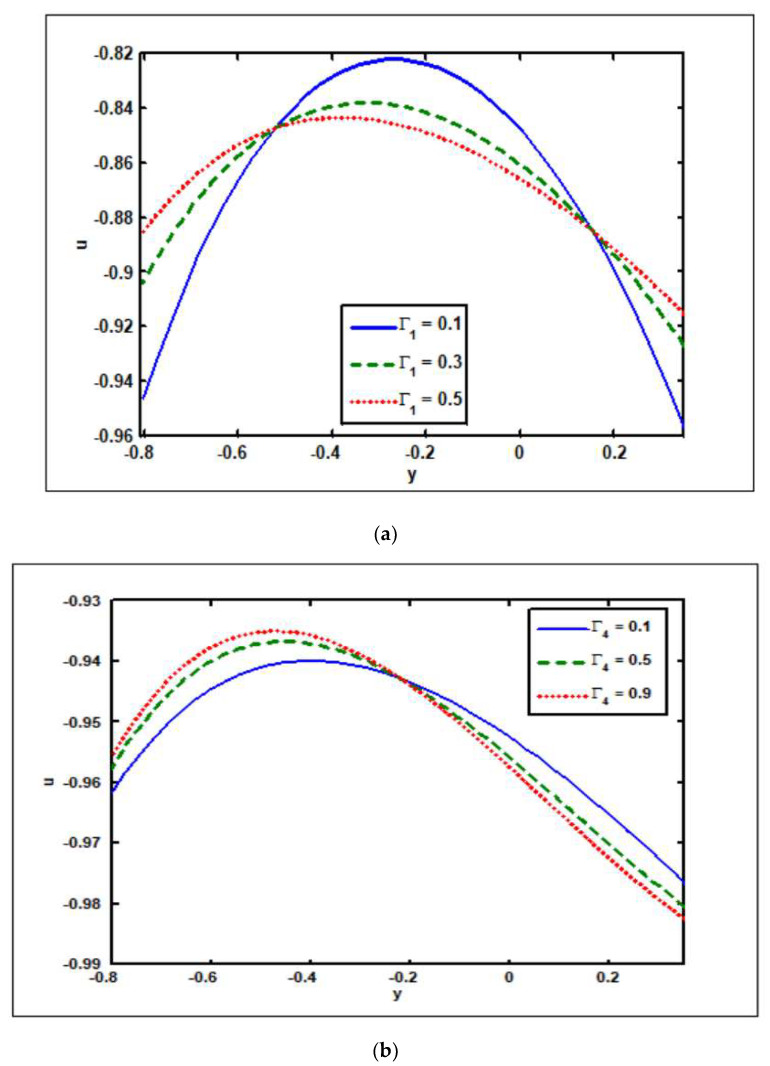

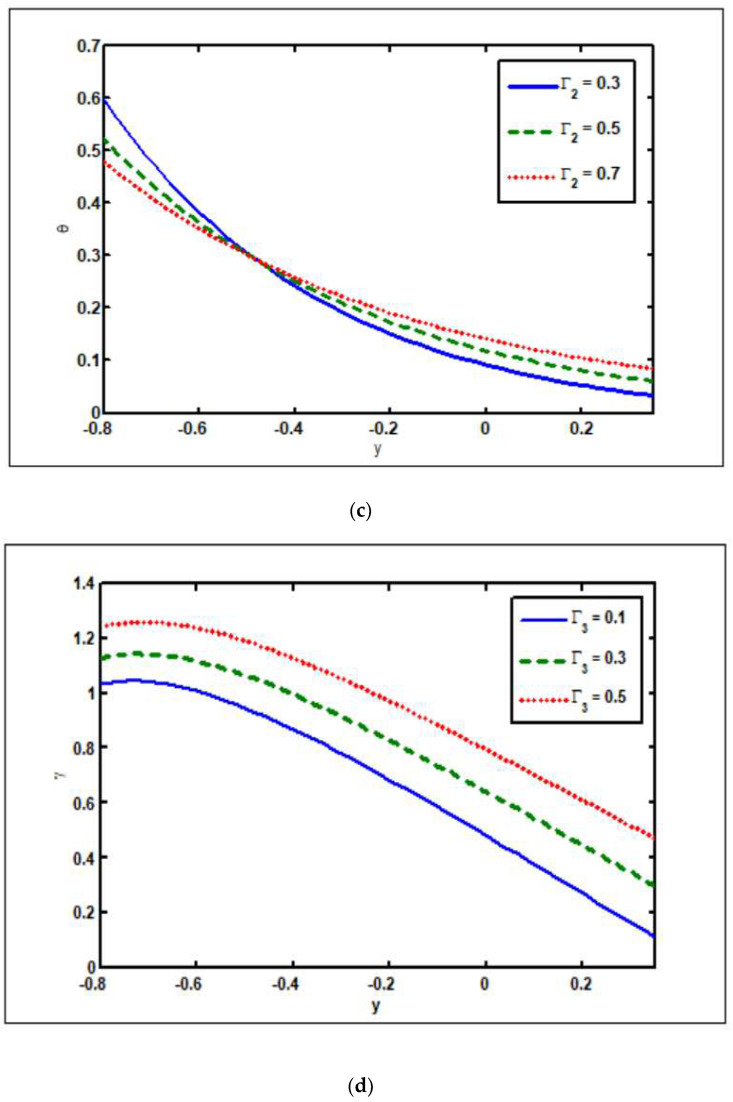

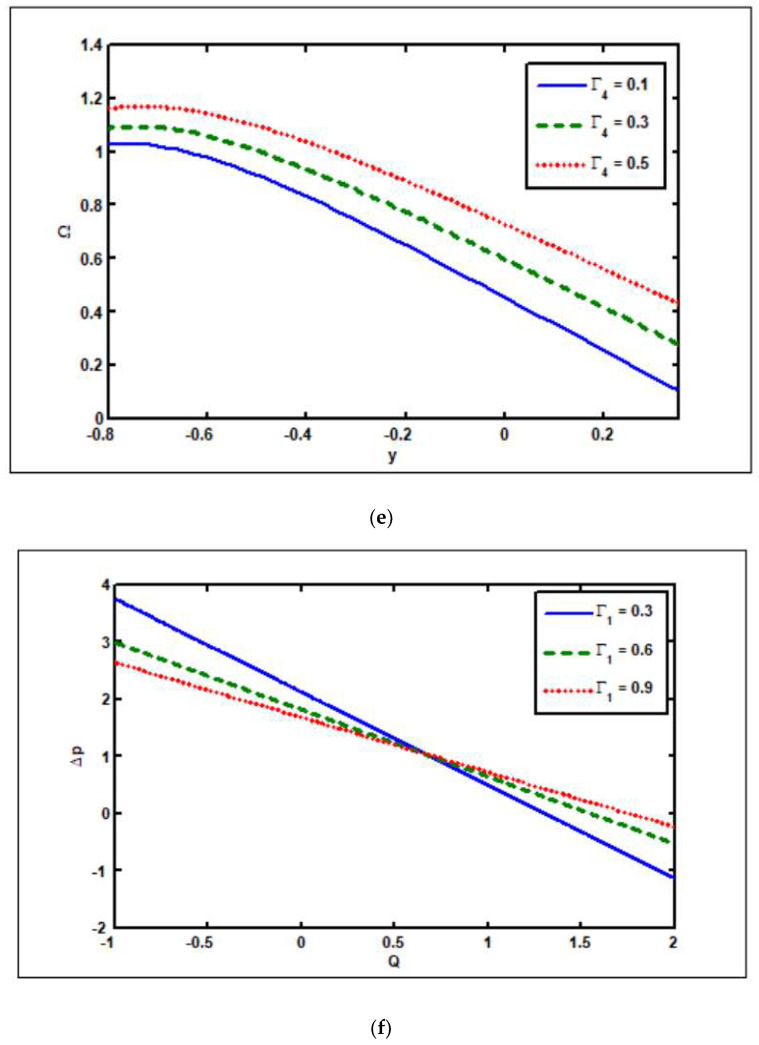

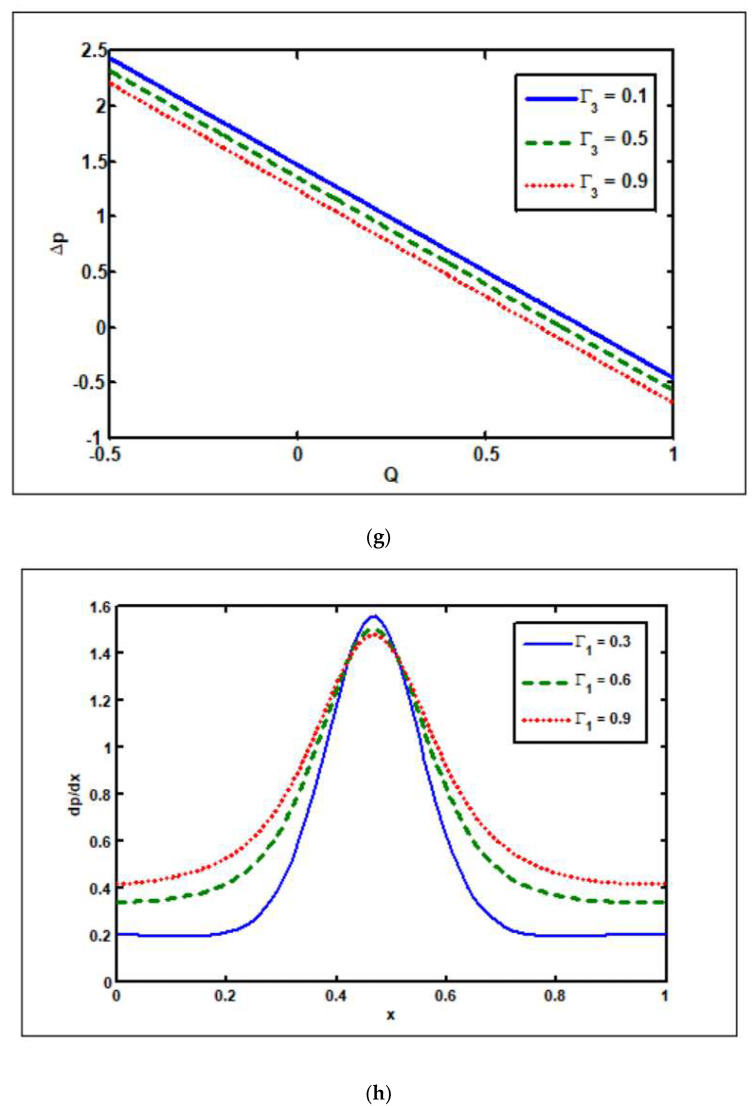

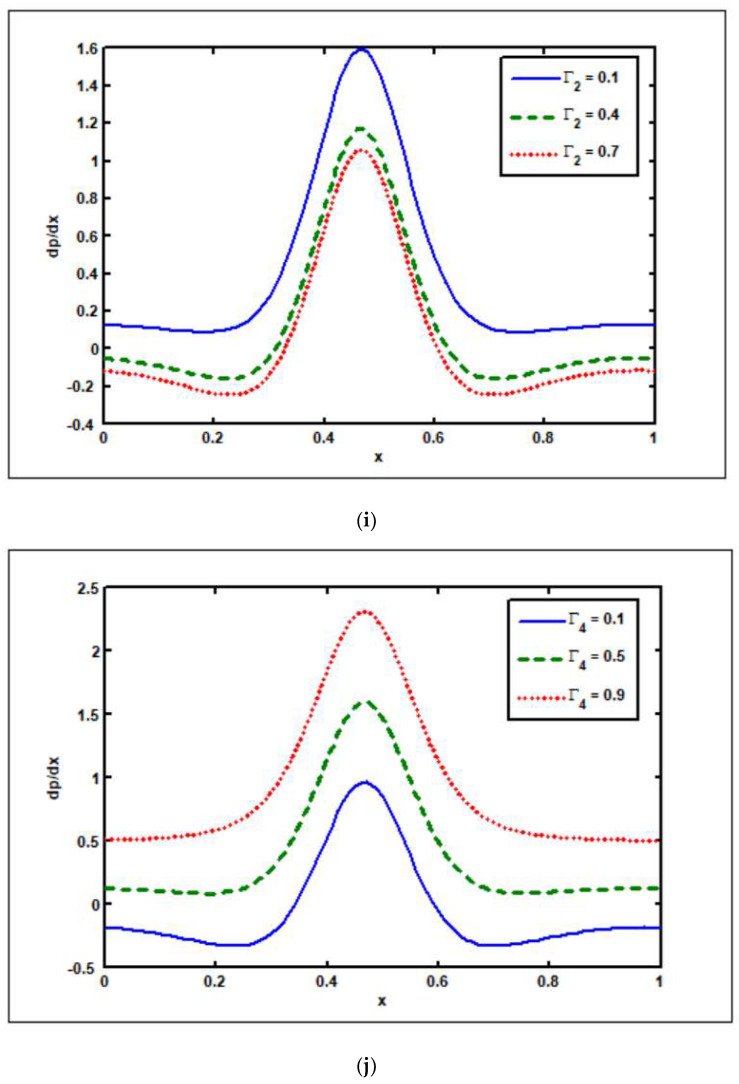
Slip parameters’ ($\Gamma_{1},\;\Gamma_{2},$ $\Gamma_{3},$ $\Gamma_{4}$ ) impact on velocity, temperature, solutal concentration, nanoparticle fraction, pressure rise, and pressure gradient. Other parameters’ values are as follows: $G_{rc}=1.1,\;N_{CT}=0.9,\;N_{TC}=1.4,\;\beta =0.1,\;M=2,\;i=2,\;G_{rF}=0.8,\;G_{rt}=0.7,\;N_{b}=0.4,\;N_{t}=0.3,\;Q=1.1,\;\omega =\pi /5,\;\varphi =\pi /4,\;a=0.8,\;b=0.3,\;d=1.1$. (**a**) $\Gamma_{2}=0.4,\;\Gamma_{3}=0.3,\;\Gamma_{4}=0.3,\;$ (**b**) $\;\Gamma_{2}=0.4,\;\Gamma_{3}=0.3,\;\Gamma_{1}=0.5,\;$ (**c**) $\Gamma_{4}=0.5,\;\Gamma_{3}=0.3,\Gamma_{1}=0.2,$ (**d**) $\;\Gamma_{4}=0.5,\;\Gamma_{2}=0.3,\;\Gamma_{1}=0.2,\;$ (**e**) $\;\Gamma_{2}=0.5,\;\Gamma_{3}=0.3,\;\Gamma_{1}=0.2,$ (**f**) $\Gamma_{2}=0.4,\;\Gamma_{3}=0.3,\;\Gamma_{4}=0.3,$ (**g**) $\Gamma_{2}=0.4,\;\Gamma_{1}=0.3,\;\Gamma_{4}=0.3,$ (**h**) $\Gamma_{2}=0.4,\;\Gamma_{3}=0.3,\;\Gamma_{4}=0.3,$ (**i**) $\Gamma_{4}=0.4,\;\Gamma_{3}=0.3,\;\Gamma_{1}=0.5,\;$ (**j**) $\Gamma_{2}=0.4,\;\Gamma_{3}=0.3,\;\Gamma_{1}=0.5$.

Figure 3a–j have been created to analyze the effect of slip factors on the velocity, temperature, solvent concentration, nanoparticle fraction, pressure rise, and pressure gradient. Figure 3a,b explain the effects of velocity on $\Gamma_{1}$ (slip parameter of velocity) and $\Gamma_{4}$ (slip parameter of nanoparticles). It is illustrated in Figure 3a that rising $\Gamma_{1}$ values increase the velocity magnitude at the channel’s center, but the opposite behavior occurs near the channel walls. In Figure 3b the velocity magnitude decreases in the region where y∈[−0.8,−0.2] due to an increase in $\Gamma_{4}$ values. Moreover, when y∈[−0.2,0.3], the velocity magnitude drops. It is seen in Figure 3c that by increasing $\Gamma_{2}$ (thermal slip parameter) values, the temperature reduces in the region where y∈[−0.8,−0.5], but when y∈[−0.5,0.3], the temperature increases. In addition, there is an opposite effect in the cases of the concentration of solute and nanoparticle fraction. The solvent concentration and nanoparticle fraction increase as the slip parameters of concentration and nanoparticles ($\Gamma_{3}$ and $\Gamma_{4}$) are increased (see Figure 3d,e). Figure 3f,g show the impact of the slip parameters of velocity and concentration ($\Gamma_{1}$ and $\Gamma_{3}$) on the pressure rise. It is worth noting in Figure 3f that raising the velocity slip parameter $\Gamma_{1}$ reduces the pressure rise in the peristaltic zone (Δp>0,Q>0), retrograde zone (Δp>0,Q<0), and free (Δp=0) pumping segments, while increasing $\Gamma_{1}$ tends to increase the pressure rise in the augmented (Q>0,Δp<0) area. It is significant to note that when enhancing the slip parameters of concentration $\Gamma_{3}$, the pressure rise in all peristaltic pumping zones (retrograde, peristaltic, free, and augmented pumping areas) tends to decrease (see Figure 3g). The effects of the slip factors of velocity, temperature, and nanoparticles ($\Gamma_{1}$, $\Gamma_{2}$ and $\Gamma_{4}$) on the pressure gradient are shown in Figure 3h,i. The pressure gradient falls at the channel’s center when the slip factors of velocity are increased, but the opposite behavior is observed near the channel walls, as shown in Figure 3h. As shown in Figure 3i, raising the temperature slip factor $\Gamma_{2}$ lowers the pressure gradient. Contrary to this, the slip factor of nanoparticles $\Gamma_{4}$ has the opposite effect (see Figure 3j).

### 3.3. Effects of Brownian Motion ($N_{b}$)

**Figure 4:**
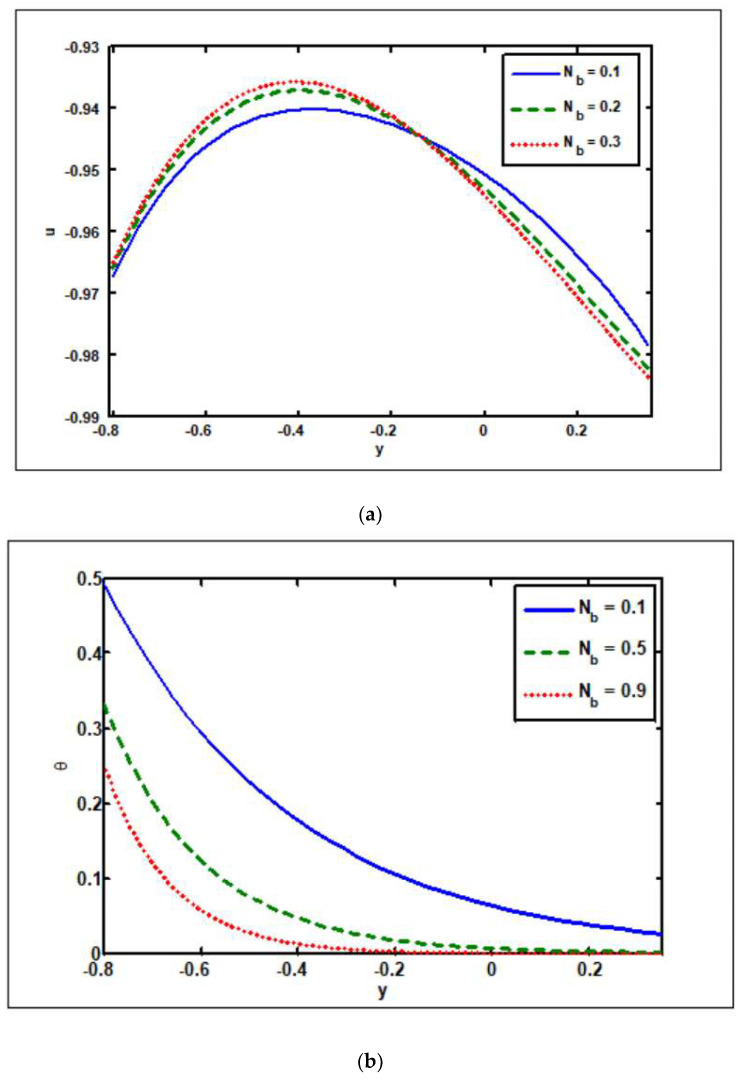

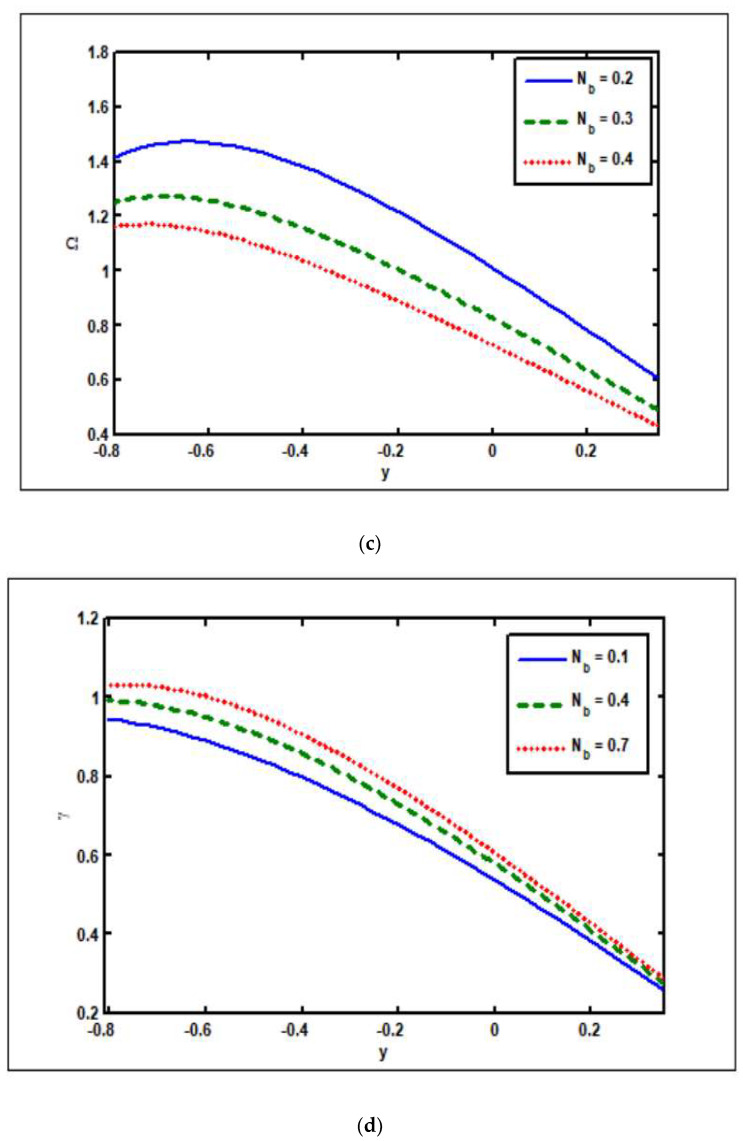

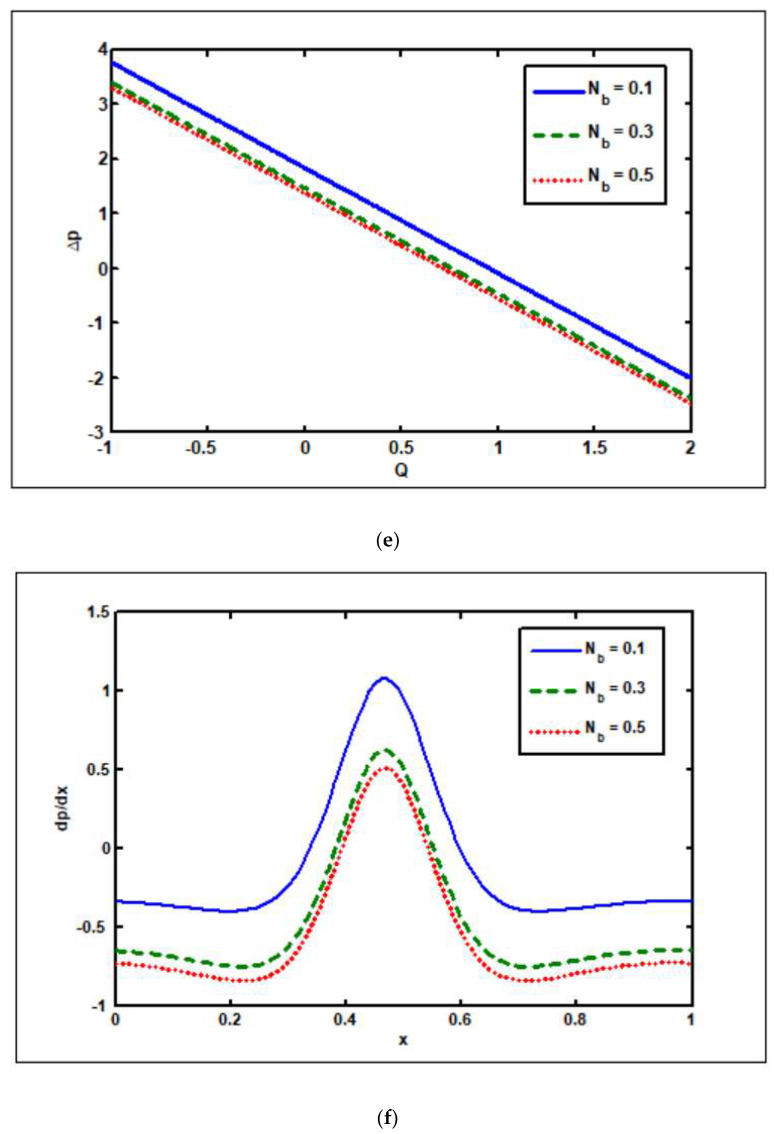
Brownian motion’s ($N_{b}$) impact on (**a**) velocity, (**b**) temperature, (**c**) nanoparticle fraction, (**d**) solutal concentration, (**e**) pressure rise, and (**f**) pressure gradient. Other parameters’ values are as follows: $G_{rc}=1.4,\;N_{CT}=0.9,\;N_{TC}=1.2,\;\beta =0.1,\;M=2,\;\Gamma_{2}=0.4,\;\Gamma_{3}=0.3,\;i=2,\;\Gamma_{4}=0.3,\;\;G_{rF}=0.8,\;G_{rt}=0.5,\;M=2.0,\;N_{t}=0.3,\;Q=1,\;\omega =\pi /5,\;\varphi =\pi /4,\;a=0.8,\;b=0.3,\;d=1.1$.

The Brownian motion’s, $N_{b}$, impacts on the axial velocity, temperature, nanoparticle fraction, pressure rise, solvent concentration, and pressure gradient are assessed in Figure 4a–f. Figure 4a demonstrates the Brownian motion’s impact on the velocity profile. As noticed in Figure 4a, the magnitude value of the velocity declines in the region where y∈[−0.8,−0.2], i.e., opposes the backflow. As a result, the Brownian motion actually slows down the flow, whereas the situation is reversed when y∈[−0.2,0.3]. The velocity magnitude rises in the given area as the Brownian motion parameter rises. It is worth noting in Figure 4b–d that increasing the Brownian motion parameter values lowers the temperature and nanoparticle fraction profiles (see Figure 4b,c), whereas increasing the Brownian motion parameter values raises the solvent concentration profile (see Figure 4d). Figure 4e depicts the role of Brownian motion on the pressure rise. The pressure rise drops in all peristaltic pumping zones as the values of the Brownian motion increase (see Figure 4e). The consequences of the pressure gradient on the Brownian motion are seen in Figure 4f. It should be noticed that as $N_{b}$ increases, the pressure gradient decreases, with the highest pressure gradient occurring at x=0.5.

### 3.4. Effects of Thermophoresis Parameter ($N_{t}$)

**Figure 5:**
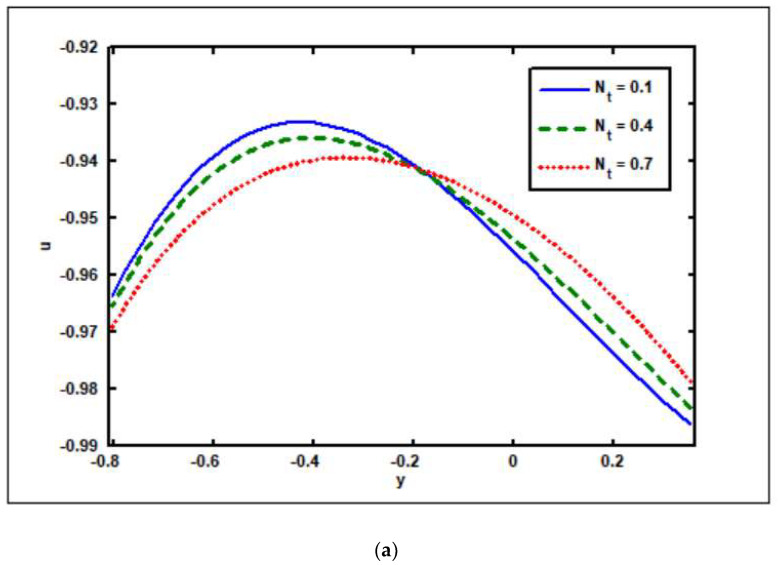

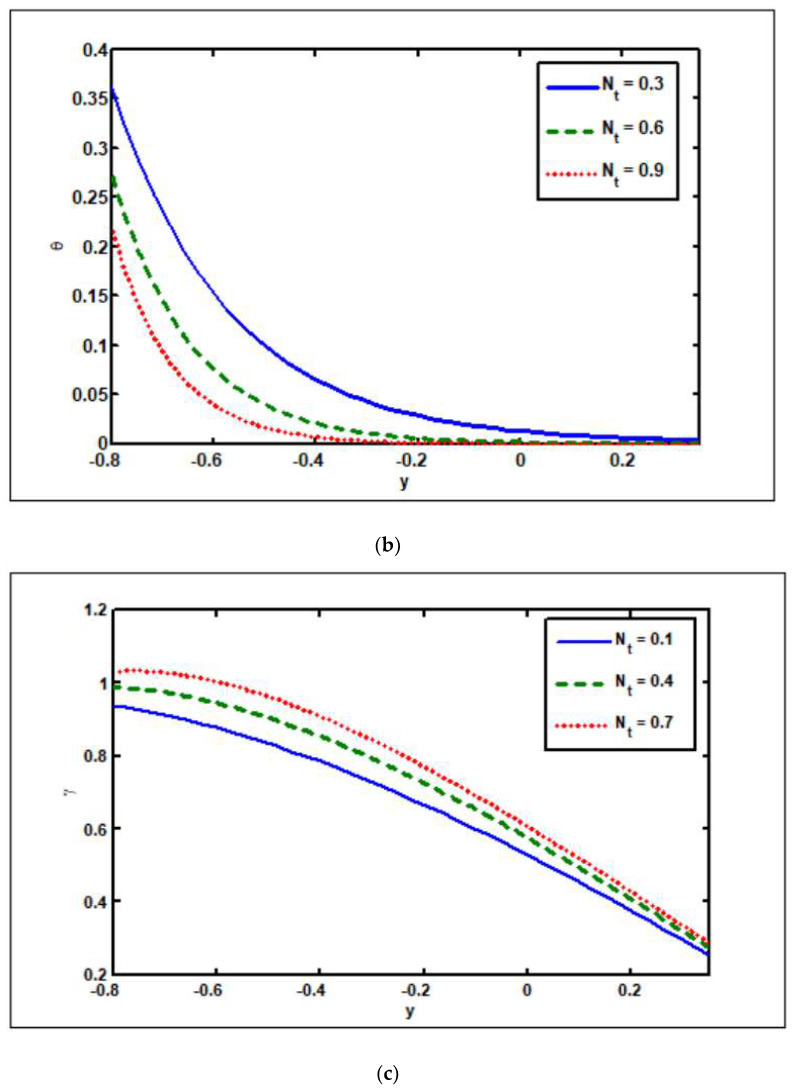

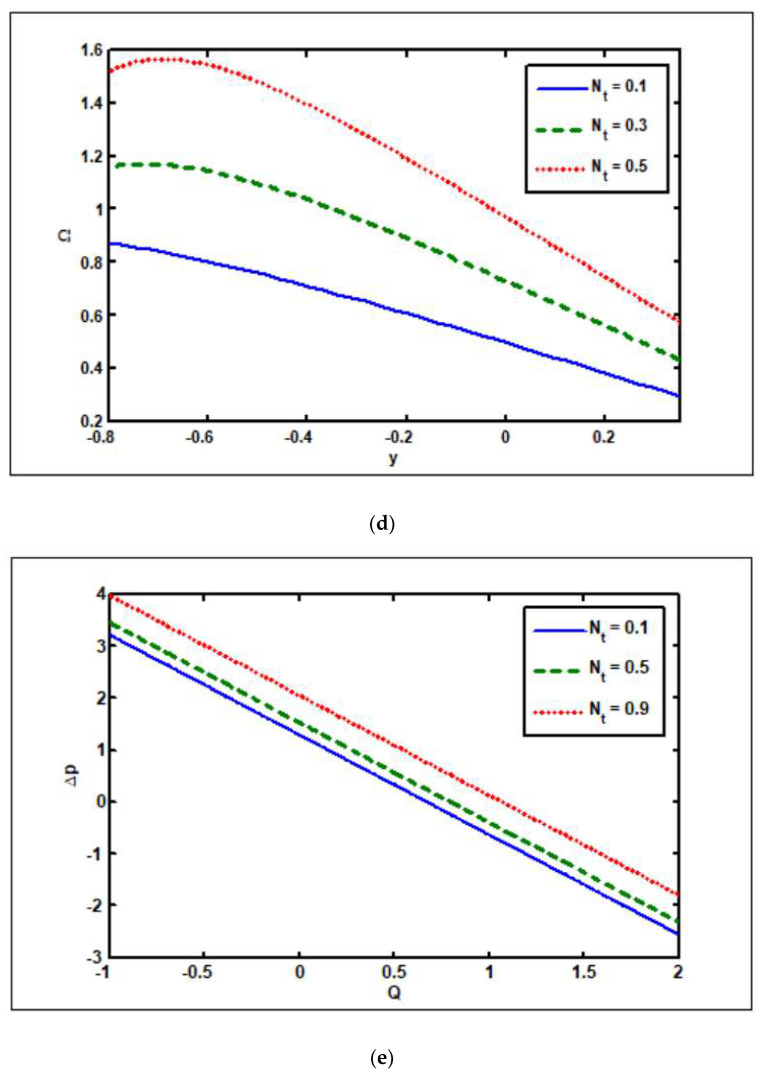

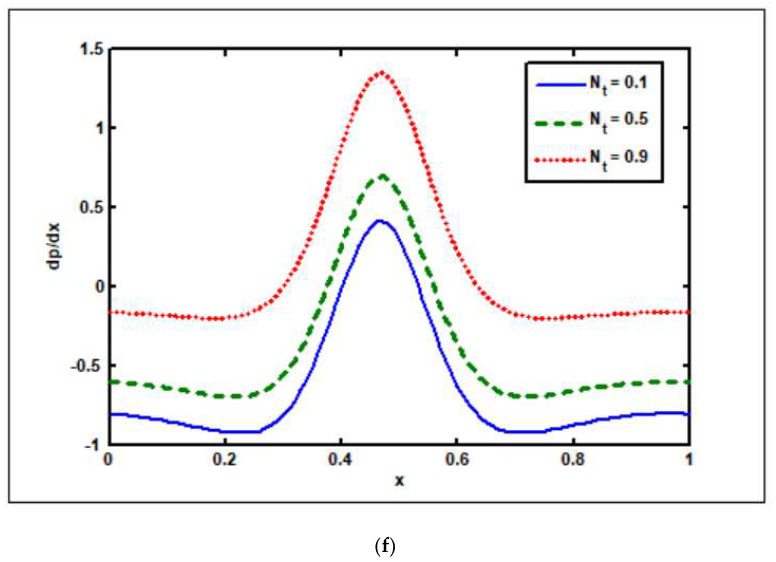
Thermophoresis ($N_{t}$) impact on (**a**) velocity, (**b**) temperature, (**c**) solutal concentration, (**d**) nanoparticle fraction, (**e**) pressure rise, and (**f**) pressure gradient. Other parameters’ values are as follows: $G_{rc}=1.4,\;N_{CT}=0.9,\;N_{TC}=1.2,\;\beta =0.1,\;M=2,\;\Gamma_{1}=0.2,\;\Gamma_{2}=0.4,\;\Gamma_{3}=0.3,\;i=2,\;\Gamma_{4}=0.5,\;G_{rF}=0.8,\;G_{rt}=0.5,\;M=2.0,\;N_{b}=0.4,\;Q=1,\;\omega =\pi /5,\;\varphi =\pi /4,\;a=0.8,\;b=0.3,\;d=1.1,\;x=0.4$.

Figure 5a–f are shown to illustrate the effects of the thermophoresis $\left(N_{t}\right)$ parameter on the axial velocity, temperature, concentration, pressure rise, nanoparticle fraction, and pressure gradient. Figure 5a depicts the velocity curve for $N_{t}$ values. The velocity magnitude grows when the fluid moves in the region y∈[−0.8,−0.2] due to the growing $N_{t}$ parameter, whereas the opposite effect is noted when the fluid moves in the region y∈[−0.2,0.3]. Here, with the rise in the thermophoresis ($N_{t}$) parameter, the magnitude of the velocity drops. Thermophoresis is a valuable source, which is caused by a temperature difference between the hot gas and the cold objects. It also controls particle migration to the cold wall. It must be noted that the temperature distribution varies as the thermophoresis parameter varies. The plots in Figure 5b show that temperature drops as the thermophoresis parameter $N_{t}$ rises. However, in the case of the concentration and nanoparticle fraction, the opposite effect has been demonstrated. In this scenario the temperature rises as the thermophoresis parameter $N_{t}$ rises (see Figure 5c,d). Figure 5e reveals the visual aspect of the pressure rise on the thermophoresis $\left(N_{t}\right)$ parameter. Increasing the thermophoresis parameter enhances the pressure rise in all peristaltic pumping zones, as seen in Figure 5e. The impact of the pressure gradient on thermophoresis ($N_{t}$) is depicted graphically in Figure 5f. As seen in Figure 5f, the pressure gradient widens as the thermophoresis parameter rises.

### 3.5. Effects of Nanoparticle Grashof ($G_{rF}$) and Thermal Grashof ($G_{rt}$)

**Figure 6:**
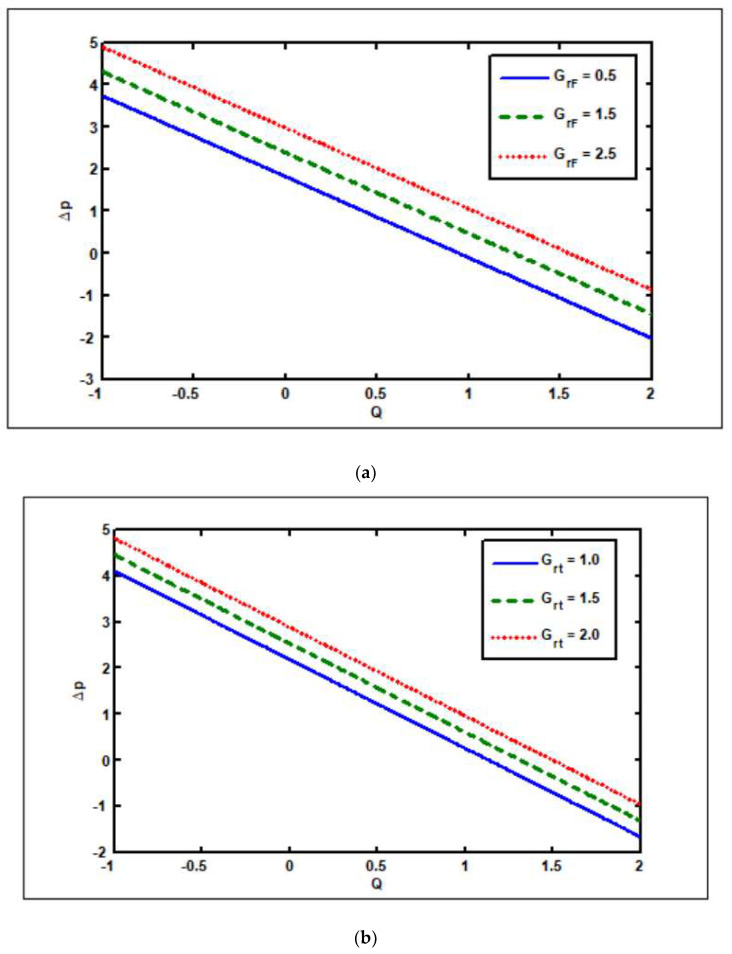

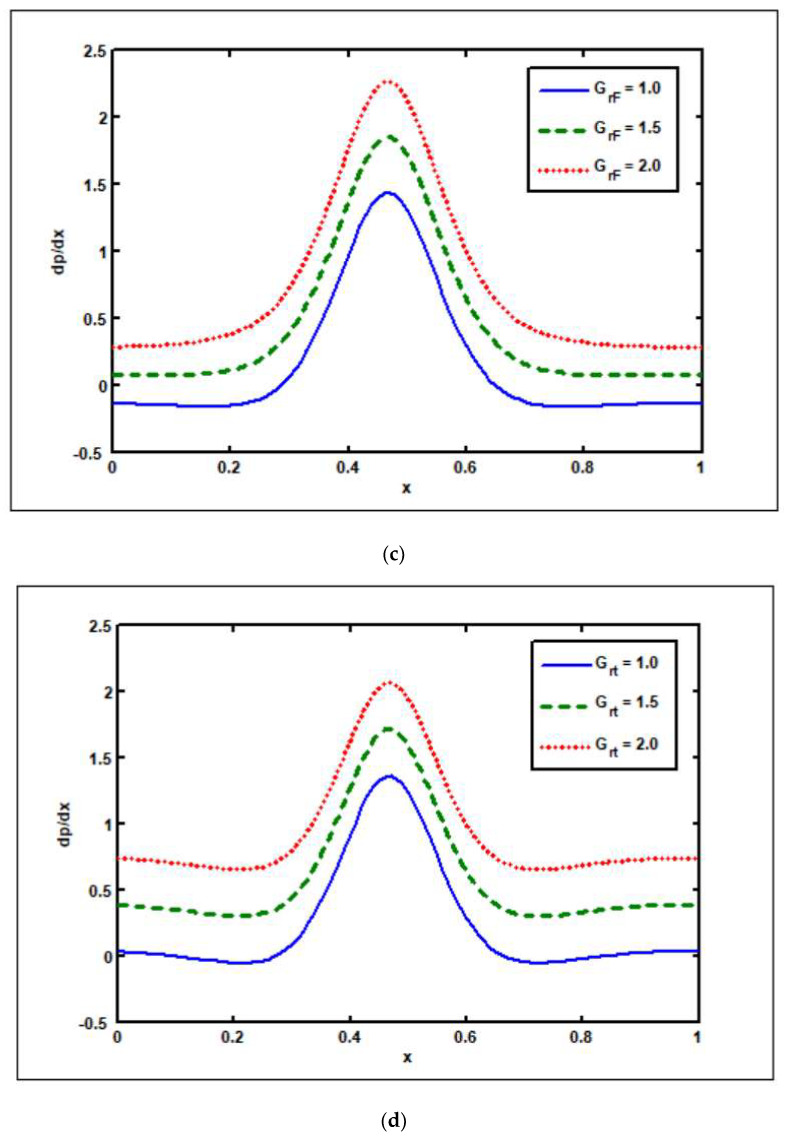

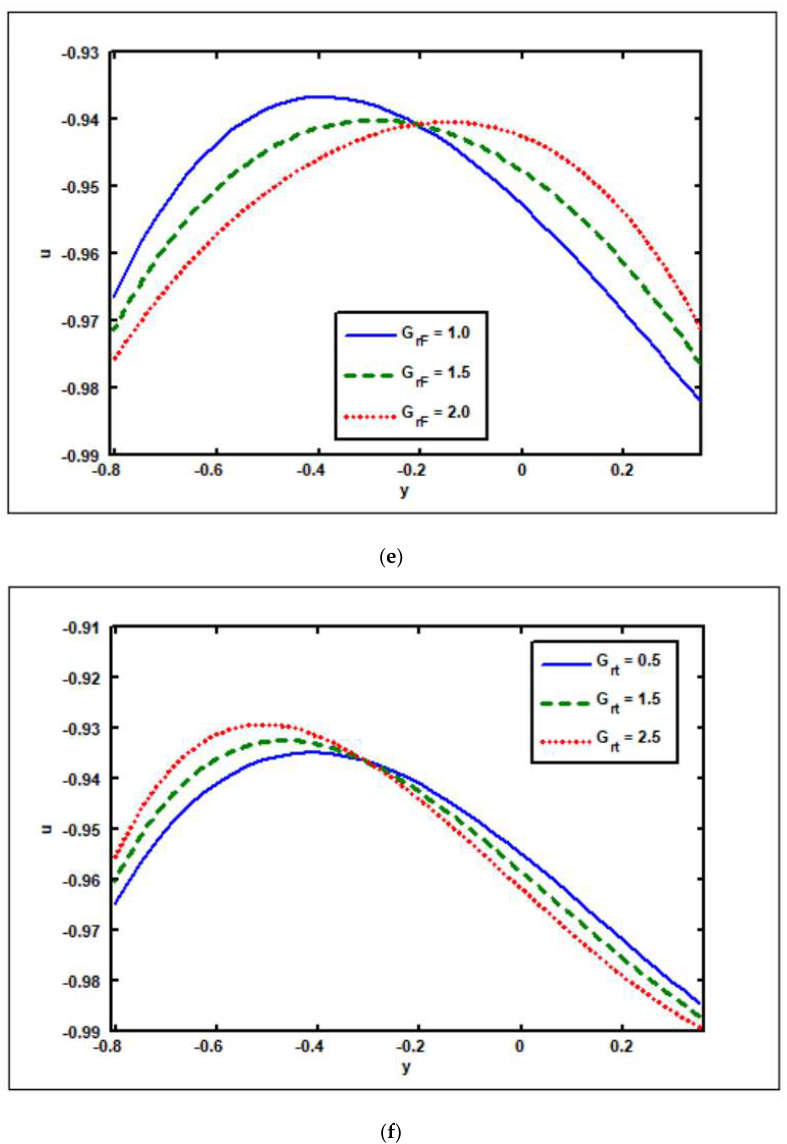
Thermal Grashof ($G_{rt}$), nanoparticle Grashof ($G_{rF}$) impacts on pressure rise, pressure gradient, and velocity. Other parameters’ values are as follows: $N_{CT}=0.9,\;N_{TC}=1.4,\;\beta =0.1,\;M=1,\;i=2,\;N_{b}=0.3,\;N_{t}=0.8,\;Q=1.0,\;\omega =\frac{\pi }{5},\;\varphi =\frac{\pi }{4},\;a=0.8,\;b=0.3,\;d=1.1,\;x=0.4,\;\Gamma_{2}=0.1,\;\Gamma_{3}=0.3,\;\Gamma_{4}=0.5,\;\Gamma_{1}=0.2$. (**a**) $G_{rt}=0.4,\;G_{rc}=0.8$. (**b**) $G_{rF}=0.4,\;G_{rc}=0.8$ (**c**) $G_{rt}=0.4,\;G_{rc}=0.8$ (**d**) $G_{rF}=0.4,\;G_{rc}=0.8$, (**e**) $G_{rt}=0.4,\;G_{rc}=0.8$ (**f**) $G_{rF}=0.4,\;G_{rc}=0.8$.

Figure 6a–f show the effects of the nanoparticle and thermal Grashof numbers on flow quantities. Figure 6a,b show the effects of the pressure rise on the nanoparticle and thermal Grashof numbers. Figure 6a,b show that increasing the number of nanoparticles Grashof $\left(G_{rF}\right)$ and thermal Grashof $\left(G_{rt}\right)$ increases the pressure rise in all peristaltic pumping zones. Figure 6c,d depict the roles of the nanoparticles and thermal Grashof numbers on the pressure rise. These graphs show that raising $G_{rF}$ and $G_{rt}$ leads to an increase in the pressure gradient. In Figure 6e, the role of the nanoparticle Grashof ($G_{rF}$) is investigated. Figure 6e illustrates that as $G_{rF}$ increases, the velocity magnitude tends to rise in the region y∈[−0.8,−0.1], whereas velocity shows the opposite tendency in the region y∈[−0.1,0.3]. The influence of the thermal Grashof number ($G_{rt}$) on velocity is seen in Figure 6f. The marginal effect of the thermal buoyancy force and viscous hydrodynamic force is represented by this parameter. The viscous forces dominate the peristaltic regime for $G_{rt}<1$, while the viscous forces dominate the peristaltic regime for $G_{rt}>1$. As the thermal Grashof number rises, the velocity magnitude drops in the region y∈[−0.8,−0.1] and vice versa for y∈[−0.1,0.3]. In most cases, thermal buoyancy tends to slow down the flow throughout the regime.

### 3.6. Trapping Effects

**Figure 7:**
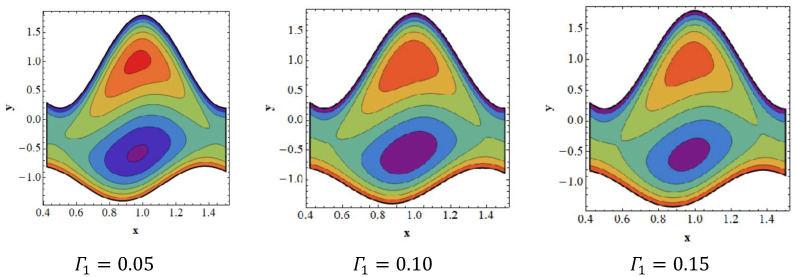
Streamlines for slip parameter of velocity $\Gamma_{1}.$ Other parameters’ values are as follows: $N_{CT}=0.9,\;N_{TC}=1.4,\;\beta =0.1,\;M=1,\;i=2,\;N_{b}=0.3,\;N_{t}=0.8,\;Q=1.0,\;\omega =\frac{\pi }{5},\;\varphi =\frac{\pi }{4},\;a=0.8,\;b=0.3,\;d=1.1,\;G_{rF}=0.4,\;\Gamma_{2}=0.1,\;\Gamma_{3}=0.3,\;\Gamma_{4}=0.5,\;G_{rt}=0.4,\;G_{rc}=0.8$.

**Figure 8:**
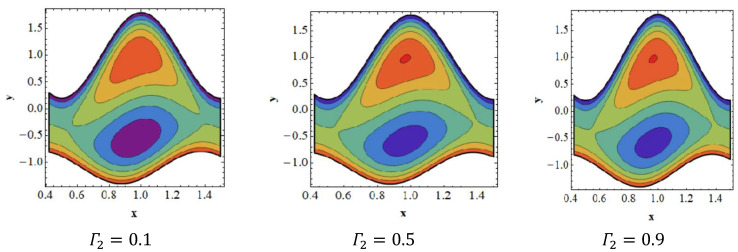
Streamlines for slip parameter of temperature $\Gamma_{2}.$ Other parameters’ values are as follows: $N_{CT}=0.9,\;N_{TC}=1.4,\;\beta =0.1,\;M=1,\;i=2,\;N_{b}=0.3,\;N_{t}=0.8,\;Q=1.0,\;\omega =\frac{\pi }{5},\;\varphi =\frac{\pi }{4},\;a=0.8,\;b=0.3,\;d=1.1,\;G_{rF}=0.4,\;\Gamma_{1}=0.1,\;\Gamma_{3}=0.3,\;\Gamma_{4}=0.5,\;G_{rt}=0.4,\;G_{rc}=0.8$.

**Figure 9:**
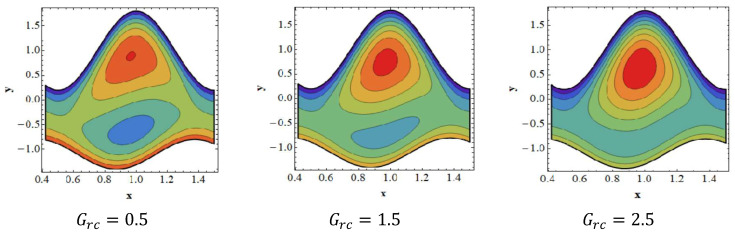
Streamlines for solutal Grashof number $G_{rc}.$ Other parameters’ values are as follows: $N_{CT}=0.9,\;N_{TC}=1.4,\;\beta =0.1,\;M=1,\;i=2,\;N_{b}=0.3,\;N_{t}=0.8,\;Q=1.0,\;\omega =\frac{\pi }{5},\;\varphi =\frac{\pi }{4},\;a=0.8,\;b=0.3,\;d=1.1,\;G_{rF}=0.4,\;\Gamma_{2}=0.1,\;\Gamma_{3}=0.3,\;\Gamma_{4}=0.5,\;G_{rt}=0.4,\;\Gamma_{1}=0.8$.

**Figure 10:**
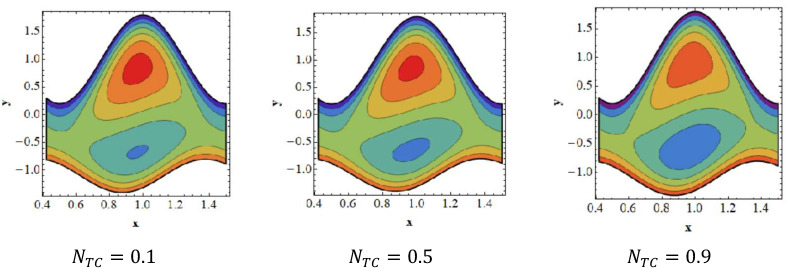
Streamlines for Dufour parameter $N_{TC}.$ Other parameters’ values are as follows: $N_{CT}=0.9,\;G_{rc}=1.4,\;\beta =0.1,\;M=1,\;i=2,\;N_{b}=0.3,\;N_{t}=0.8,\;Q=1.0,\;\omega =\frac{\pi }{5},\;\varphi =\frac{\pi }{4},\;a=0.8,\;b=0.3,\;d=1.1,\;G_{rF}=0.4,\;\Gamma_{2}=0.1,\;\Gamma_{3}=0.3,\;\Gamma_{4}=0.5,\;G_{rt}=0.4,\;\Gamma_{1}=0.8$.

Trapping seems to be a remarkable phenomenon of propelled peristaltic flows. Trapping occurs when the inner fluid moves, which creates a mass along with a peristaltic wave streamline. Streamlines grab a fluid mass bolus to drive it forward with peristaltic waves at high flow rates and substantial occlusions. To investigate the phenomenon of streamlines, Figure 7, Figure 8, Figure 9 and Figure 10 are drawn. Figure 7 shows a decrease in the trapped bolus volume of the lower part of the channel, whereas the amount and volume of the trapped bolus decreases in the upper half due to growing values of the slip parameter of velocity $\Gamma_{1}.$ In Figure 8 it is noted that due to growing values of the slip parameter of temperature $\Gamma_{2}$, the quantity and size of the trapped bolus increases in the lower half of the channel, whereas the trapped bolus size decreases in the upper half. From Figure 9 it is illustrated that in the upper section of the channel, raising the solutal Grashof number $G_{rc}$ increases the number of trapped boluses, but the opposite impact is shown in the lower part. As seen in Figure 10, the number of boluses in the upper area of the channel decreases as the Dufour parameter is increased; however, the trapped bolus volume grows in the lower half.

## 4. Concluding Remarks

This section covers the concluding remarks on the ongoing problem. A theoretical evaluation is shown to explore the partial slip impact on the double diffusion convection of peristaltic transport in Sisko nanofluids along an asymmetric channel by taking an inclined magnetic field. To understand the flow dynamics of the current problem, mathematical modeling is considered. Numerical solutions are proposed for the problem under analysis. The key findings are as follows:

- The temperature and nanoparticle profiles drop as the Brownian motion is increased, while the concentration profile rises.
- The velocity magnitude at the channel’s center grows as the slip factor of velocity ($\Gamma_{1}$) increases, but the opposite behavior occurs at the channel walls.

- As the slip parameters of concentration and nanoparticles are enhanced, the solvent concentration and nanoparticle fraction increase.
- The temperature drops as the thermophoresis parameter rises.
- The amount of bolus in the upper channel decreases as the Dufour parameter is increased; however, the volume of the trapped bolus grows in the bottom half.
- The trapped bolus grows in terms of size and number in the bottom half of the channel as the slip parameter of temperature increases, while the size of the trapped bolus decreases in the top half.

## Data Availability

Not applicable.

## Nomenclature

T	Temperature
Pr	Prandtl number
C	Solutal concentration
M	Hartmann number
Ω	Nanoparticle volume fraction
$N_{b}$	Brownian motion parameter
Re	Reynolds number
$G_{rt}$	Thermal Grashof number
$G_{rF}$	Nanoparticle Grashof number
Le	Lewis number
$N_{t}$	Thermophoresis parameter
Ln	Nanofluid Lewis number
${\left(\rho c\right)}_{f}$	Heat capacity of fluid
${\left(\rho c\right)}_{p}$	Heat capacity of nanoparticle
$N_{CT}$	Soret parameter
$D_{TC}$	Dufour diffusively
$D_{B}$	Brownian diffusion coefficient
$D_{T}$	Thermophoretic diffusion coefficient
$D_{s}$	Solutal diffusively
$N_{TC}$	Dufour parameter
$G_{rc}$	Solutal Grashof number
$D_{CT}$	Soret diffusively
**Small alphabets**	
u	Axial velocity
v	Transverse velocity
k	Thermal conductivity
t	Time
${\bar{a}}_{1},\;{\bar{a}}_{3}$	Channel width
p	Pressure
g	Acceleration due to gravity
b	Wave amplitude
${\bar{a}}_{2},$ ${\bar{a}}_{4}$	Wave amplitudes
c	Propagation of velocity
**Greek symbols**	
$\rho_{f}$	Fluid density
$\rho_{f_{0}}$	Fluid density at $T_{0}$
$\rho_{p}$	Nanoparticle mass density
${\left(\rho c\right)}_{p}$	Nanoparticle heat capacity
$\beta_{T}$	Volumetric coefficient of thermal expansion
$\beta_{C}$	Volumetric coefficient of solutal expansion
δ	Wave number
Θ	Temperature
$\Gamma_{1}$	Velocity slip parameter
$\Gamma_{2}$	Temperature slip parameter
$\Gamma_{3}$	Concentration slip parameter
$\Gamma_{4}$	Nanoparticles slip parameter
δ	Wavelength
Ψ	Stream function
γ	Solutal concentration
ω	Magnetic field inclination angle
